# A Promising Review on Cyclodextrin Conjugated Paclitaxel Nanoparticles for Cancer Treatment

**DOI:** 10.3390/polym14153162

**Published:** 2022-08-03

**Authors:** Kamini Velhal, Sagar Barage, Arpita Roy, Jaya Lakkakula, Ramesh Yamgar, Mohammed S. Alqahtani, Krishna Kumar Yadav, Yongtae Ahn, Byong-Hun Jeon

**Affiliations:** 1 Amity Institute of Biotechnology, Amity University, Mumbai-Pune Expressway, Bhatan, Panvel, Mumbai 410206, India; kaminivelhal@gmail.com (K.V.); shbarage@mum.amity.edu (S.B.); 2 Centre for Computational Biology and Translational Research, Amity Institute of Biotechnology, Amity University, Mumbai-Pune Expressway, Bhatan, Panvel, Mumbai 410206, India; 3 Department of Biotechnology, School of Engineering & Technology, Sharda University, Greater Noida 201310, India; arbt2014@gmail.com; 4 Department of Chemistry, Chikitsak Samuha’s Patkar-Varde College of Arts, Science and Commerce, Goregaon (West), Mumbai 400104, India; rameshyamgar@gmail.com; 5 Radiological Sciences Department, College of Applied Medical Sciences, King Khalid University, Abha 61421, Saudi Arabia; mosalqhtani@kku.edu.sa; 6 BioImaging Unit, Space Research Centre, Michael Atiyah Building, University of Leicester, Leicester LE1 7RH, UK; 7 Research Center for Advanced Materials Science (RCAMS), King Khalid University, Abha 61413, Saudi Arabia; 8 Faculty of Science and Technology, Madhyanchal Professional University, Ratibad, Bhopal 462044, India; envirokrishna@gmail.com; 9 Department of Earth Resources & Environmental Engineering, Hanyang University, 222-Wangsimni-ro, Seongdong-gu, Seoul 04763, Korea; ytahn83@hanyang.ac.kr

**Keywords:** paclitaxel PTX, cyclodextrin CD, nanoparticles NPs, novel drug delivery system NDDS

## Abstract

This review presented the unique characteristics of different types of cyclodextrin polymers by non-covalent host–guest interactions to synthesize an inclusion complex. Various cancers are treated with different types of modified cyclodextrins, along with the anticancer drug paclitaxel. PTX acts as a mitotic inhibitor, but due to its low dissolution and permeability in aqueous solutions, it causes considerable challenges for drug delivery system (DDS) designs. To enhance the solubility, it is reformulated with derivatives of cyclodextrins using freeze-drying and co-solvent lyophilization methods. The present supramolecular assemblies involve cyclodextrin as a key mediator, which is encapsulated with paclitaxel and their controlled release at the targeted area is highlighted using different DDS. In addition, the application of cyclodextrins in cancer treatment, which reduces the off-target effects, is briefly demonstrated using various types of cancer cell lines. A new nano-formulation of PTX is used to improve the antitumor activity compared to normal PTX DDS in lungs and breast cancer is well defined in the present review.

## 1. Introduction

Cancer is unregulated malignant cell growth, which can infect different parts of body. There are different types of the cancers, such as drug-resistant ovarian, metastatic breast, lung cancer, prostate, blood cancer, etc. There are multiple steps involved in the treatment that depend on the cancer stage, which include radiation, surgery and chemotherapy or it may be a combination of both along with surgery. These treatments cause several adverse effects, leading to the damage of normal tissue and organs along with cancer infected cells. As per the WHO survey, in the near future, morbidity and mortality will increase due to cancer [1]. The goal is to derive a system that has the desired drug loading capacity, a long shelf-life and lower toxic effects.

Nanotechnology has attracted more interest in recent years, as others wish to about learn the interaction between nanomaterials with the biological environment and to obtain productive DDS. The system of delivering antibiotic drugs to the desired region of the tumor for enhanced therapeutic effect is called DDS [2]. However, some drugs, such as paclitaxel, doxorubicin and curcumin, have great potential to kill the cancer cells with severe side effects. These properties hamper the targeted drug delivery and induce off-target effects. To circumvent the limitations, alternative strategies have been explored. Existing drugs with modified formulations are gaining more attention for the desired effect. In this context, different types of polymers have been explored, which can be used as a carrier, such as Genexol-PM, which is used with polymeric micelles for paclitaxel encapsulation composed of PEG-(d,l-lactide). It is the first polymeric micelle formulation with approval from the FDA [3]. Another naturally occurring biodegradable polymer is chitosan, which is obtained from the deacetylation of chitin and has been used as a pH-based delivery system [4]. Overall, polymer (PLGA/chitosan/PVA/alginate) drug delivery would ensure the delivery of therapeutics to the targeted site and maintain drug concentration within the polymer for the desired duration. It ensures that the molecules target the impacted organs and prevent side effects on healthy tissues. 

As with polymers, nanoparticles show unique properties, such as unique optical, magnetic, electrical, thermal behavior and a high surface-volume ratio. These are solid particles that range between 10 and 100 nm. Different types of NPs (CD/C6 NVs, C6 NPs and CD/ NP (C6) NVs, FCD-1 and FCD-2 NPs, HO-CNPs, tripalm-NPs, CS NPs, etc.) help to target a specific site for the delivery of therapeutic substances. Nowadays, different varieties of nanocarriers have been developed against cancer cells [5,6,7]. Polymeric nanoparticles (NPs) play an excellent role in diagnostic and therapeutic applications, such as bioavailability improvements and controlled release of the drugs. Various biodegradable polymers that are utilized in NP formulations are polyesters, polyanhydrides, polyamides, polysaccharides, polycaprolactone, polyglycolic acid, polylactic acid, polypeptides, etc. [8], which are used as a delivery vehicle and enhance the solubility of the hydrophobic drugs. In the present review, we focus on hydrophobic drugs that show low permeability, solubility, and severe side effects. For example, PTX is used as an antitherapeutic drug to treat persons with different types of cancers, such as lungs, ovarian, breast etc. [9]. Paclitaxel (PTX) belongs to class-IV in the BCS, which shows low permeability and aqueous solubility, thus possessing substantial challenges for designing an effective delivery system. PTX acts as a mitotic inhibitor, a natural diterpenoid anticancer drug. The solubilization of paclitaxel to form an inclusion complex using cyclodextrin improves its physicochemical properties [10]. Wang et al. prepared PTX DDS using chitosan and sodium alginate to modify PLGA(poly-lactic-co-glycolic-acid) NPs through layered deposition that formed three-layer nanoparticles (PLGA/CS/ALG). This was developed in order to reduce the burst effect and increase the encapsulation efficiency for PTX. Additonally, results from in vitro, cellular uptake and MTT assays in HepG-2 cellsrdepicted controlled drug release with enhanced cytotoxicity and stability. Hence, the developed delivery system was considered to be having an excellent potential as an NDDS [11].

More recently, cyclodextrin NPs prepared using amphiphilic β-CD, along with 6C(6-*O*-CAPRO-β–CD) and loaded with PTX (paclitaxel), were found to be more stable for 1 month, which significantly reduces the hemolytic properties resulting from the commercial vehicle (Cremophor-EL) [12,13]. The amphiphilic CD can form a nanosphere or nano-capsule by a simple nanoprecipitation technique. 

**Figure 1:**
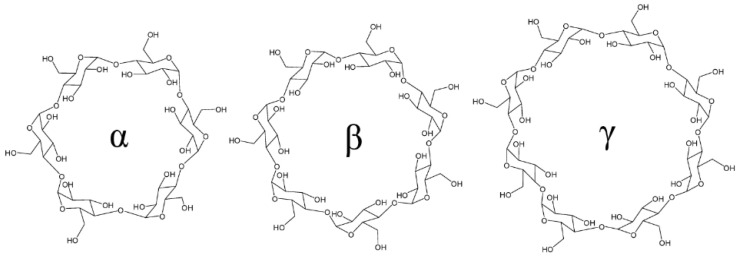
Structure of three parent cyclodextrin. (Adapted with permission from Ref. [14]. Copyright © 2009 American Chemical Society).

Cyclodextrin is important to increase the solubility and permeability of the anticancer drugs. Among various types of polymers studied, cyclodextrin (CD) has a unique toroidal shaped structure with hydrophilic outer surface and internal hydrophobic cavity. When cyclodextrins interact with appropriately sized molecules, a non-covalent inclusion complex is formed. The advantage of this inclusion complex over the hydrophobic drugs is that it shows improved solubility and stability. Naturally found CDs have a glucopyranose α- (six units), β- (seven units), and γ- (eight units) forming cyclic structure (Figure 1). CD derivatives have been used due to their low toxicity and high biocompatibility [14]. They are studied for boosting the anticancer drugs’ properties. The CDs are used in the prepared supramolecular delivery system with different varieties of drug molecules. The binding process of CDs with drugs modify the physicochemical and biological properties. 

The present review emphasizes various CD-conjugated paclitaxel nanoparticles for their efficacy, shape, size, cytotoxic activity and ability to kill different types of cancer cells using a novel delivery system. The main focus of the review is to outline the important findings and applications of cyclodextrins for treating several types of cancer. 

## 2. Cyclodextrin Nanoparticles for Anticancer Application

PTX drugs have been extensively applied in tumor treatment of various carcinomas, e.g., ovarian, lungs, breast, etc. Although, it’s therapeutic applications are restricted by low water solubility. To address the problem of enhancing PTX water solubility, one of the encouraging approaches is to form an IC using CDs and derivatives for significant results. The attractive part of the CD is its structure containing 7-d glucose attached by R-1 and 4-glucose acts as host for DDS due to its molecular size, superb biocompatibility, outer surface, which is hydrophilic, and internal cavity, which is hydrophobic in nature [15]. Therefore, CD and their derivatives act as a nanobot to reduce adverse side effects caused during cancer therapy and sustained release of the drug and their solubility in different types of the cancers are presented in the present review.

### 2.1. Cyclodextrin Nanoparticles Used for Breast Cancer Treatment

The intention of any anticancer therapy is to destroy only cancer cells without damaging the normal cells. The most rapidly growing cancer with high mortality rates among women worldwide is breast cancer (BC). As per the 2018 American survey, breast cancer was diagnosed in 266,120 women and resulted in 40,920 deaths [16].

In another experiment, the researcher aimed to increase the permeability and solubility of the PTX that can be formulated with different β-cyclodextrin derivatives. Shah et al. formulated the inclusion complex CD–PTX (1:1, 1:2, and 1:5 molar ratios PTX and CD) by the co-solvent lyophilization method. The entrapment efficiency for PTX was identified as 0.38 µg/mL, measured at 25 °C. The HNMR characteristic resonance peak that occurred around 7.5–8.5 ppm effectively shows that PTX was entrapped within CD. In vitro genotoxicity and cytotoxicity studies carried out using modified MTT assay on A2780, A549, HT-29, PC-3 and MCF-7 cell lines confirmed the safety of CDs as excipients, as the ID_50_ value killed 50% of the cells. Hence, the observed data indicated that the highest quantity of PTX was present inside the cell and improves cellular internalization and solubility of the class IV drug [17].

Nieto et al. developed a novel paclitaxel-trastuzumab-targeted transport nano vehicle for treating various cancer cells, which highlights the human epidermal growth-factor-receptor 2 (HER2). Piperazine and alginate nanoparticles (APPZ) were synthesized and encapsulated paclitaxel, forming β-CD complexes (300 nm) and trastuzumab. The sizes were recorded to be nearly 296 ± 50 nm with a superficial charge of −24 ± 1.4 mV. The internalization of APPZ in HER-2 was analyzed by immunofluorescence which occurred due to the endocytosis process and the progressive accumulation of trastuzumab was further observed in intracellular sites. Further to understand the different levels of HER2 expression an antitumor activity was studied on human cell lines (BT474, SKBR3, OVACAR3, and HS5. Interestingly, it was observed that, in comparison to free PTX: β-cyclodextrins, the conjugated nano vehicle resulted in more specifics for HER2 treatment for overexpressing cells through endocytosis. It showed that the proposed nano vehicle can improve the bioavailability of PTX and targets HER-2 positive cancer cells [18].

Aguero et al. studied the oral bio-availability of PTX on rats. Firstly, they synthesized nanoparticles by conjugating PTX and cyclodextrin at a 1:1 molar ratio, where PTX was dissolved in methanol and then added to the different CD solutions using β- CD, HPCD and NHCD. Secondly, the stock solution of PTX was added to 100 mg of poly anhydride containing 5 mL of acetone and it was stirred using a magnetic stirrer for 30 min at room temperature for the synthesis of the anhydride PTX-loaded CDs. The resulting solvent was further centrifuged to remove the supernatant and the pellets were resuspended in water and freeze dried for further use. The size of compounds, their zeta-potential, yield of compounds, amount of PTX, and EE were recorded to be (298 ± 6, −39.3 ± 5.2, 68.6 ± 4.4, 38 ± 3.1 and 28.1), (307 ± 7, −42.1 ± 1.4, 63.3 ± 2.9, 167± 8.3, 97.4) and (310 ± 6, −34.5 ± 3.9, 59.5 ± 4.6, 91 ± 7.6, 61.6) for PTX/CD, PTX/HPCD and PTX/NHCD, respectively, as observed in Table 1. It was identified that the oral bioavailability of PTX was found to be 80% for PTX/CD, PTX/HPCD and 18% for PTX/NHCD due to the presence of aminated-cyclodextrin. The plasma curve for an anticancer drug for PTX-CD is characterized by PTX concentration for 24 h after administration. The sustained release of the drug was found to be approximately 27 and 33 times higher for PTX/CD and PTX/HPCD, respectively. The combination of CD and poly(anhydride) with NPs exhibits an improvement with HPCD or CD over the oral bio-availability of PTX [19].

Jing et al. prepared a layer by layer (LbL) capsule synthesized with hyaluronic acid (HA), formed a β/CD and poly(L-lysin) (PLL) complex on calcium carbonate particles and using the host-guest mechanism, PTX was loaded LbL. The complexation of PTX was found to be 9 ± 1 from the ^1^H NMR analysis and PTX was found to induce large changes for the H-3 and H-5 protons, which are found inside the β-CD cavity. This was in contrast to the proton found outside of the cavity surface, which formed an inclusion complex. Secondly, poly (L-lysine (PLL)) colloidal particles were used to assemble an LBL capsule that combined the inclusion complex via the host–guest interaction. The amount of Oregon Green-labeled PTX within the capsule was identified using a fluorescence microplate reader and the result was found to be 550 ± 5 nm and at a 650 ± 5 nm wavelength. Under the optimum conditions, PTX was discharged in two-step processes, with about 20% of the drug release in 4 h., followed by increased controlled release up to 12 h. and with the addition of DM- β-CD, 80% of PTX was released in nearly 20 h. Furthermore, they performed a cytotoxicity study using MDA -MB-231 by MTT assay and observed that the PTX capsule affected cell lines that inhibit the proliferation and metabolic activity 7.2 to 8.6 times less compared to cells cultured without the capsule. The studies on MDA-MB-231 cells indicated that HA exhibited-controlled release in addition to constant release of PTX drug from the capsule on the targeted area that killed the breast cancer cells. Hence, HA-CD capsules showcased excellent potential in delivering hydrophobic drugs to tumor cells in a controlled manner [20].

Erdogar et al. synthesized two types of amphiphilic cyclodextrins, FCD-1 and FCD-2, using the nanoprecipitation method along the folate group that activates breast tumors that are folate positive. The main purpose was to develop and characterize new CDs via the 3^2^ factorial design. In a physicochemical characterization study performed using blanks, PTX-loaded FCD-1 and FCD-2 NPs were found to be spherical shape with a narrow unimodal size in the range of 70–275 nm and 125–185 nm, whereas the zeta potential value was recorded to be neutral and −20 mV for the nanoparticles. In a cell culture study performed using the L929 fibroblast, Zr-75-1 and T-47D concluded that no cytotoxicity was observed against the L929 cells. FCD-2 and FCD-1 NPs loaded with Nile red was applied to both breast cancer cells for 4 s. FCD-1 NPs were distributed homogenously inside cells, demonstrating nuclear and cytoplasmic localization compared to FCD-2. The anticancer efficacy of PTX-loaded Fr-targeted NPs substantially increased the Zr- 75-1 cells’ sensitivity for anticancer agents. Hence, PTX-loaded folate-conjugated CD-NPs should be considered as the most promising, safe, and effective DDS for anticancer applications [6].

Similarly, FCD-1 and FCD-2 were studied to increase the antitumor efficacy and reduce the toxicity of PTX on metastatic breast cancer. Erdogar et al. conducted an animal study using PTX-loaded FCD NPs. The characterization results for PTX/FCD- 1 and PTX/FCD-2 NPs were found to be as follows: the sizes were 84.8 nm and 161.2 nm; Zeta P was −5.1 mV and −24.5 mV; EE was 60.4% and 35.1%; PTX per mg of CD was 85.5 and 105.4. A cytotoxicity study that used 4T1 breast cancer cell lines over 48 h depicted a 60% loss of activity in PTX/FCD NPs. An antitumor efficacy study performed using PTX/FCD-1 and PTX/FCD-2 on 4T1 human breast cancer cell bearing mice showed the longest survival rate with PTX/FCD-1 NPs, as compared to PTX/FCD-2, with least weight loss in the mice treated with PTX/FCD-1. Further, histopathological evaluation revealed no damage or cell separation in the presence of PTX/FCD-1 NPs. It was found that FCD-1 NPs showed 60.4% EE compared to FCD-2 (35.1%); thus, it was concluded that PTX-loaded FCD-1 Np can serve as a potential carrier, which can allow PTX to target the tumor [7].

Baek et al. developed a PTX DDS to increase the absorption of PTX and reduce nephrotoxicity, which accompanies intravenous administration. They studied both PTX-loaded solid lipid nanoparticles (PTX/SLN) and PTX/HPCD-PSC (size 251.4 ± 12.0 nm) using the method of hot-melted sonication. Anticancer activity was studied on MCF-7 cells using Nile red solution with the help of confocal microscopy to identify the cellular uptake. The results obtained further confirmed increase in apoptosis in presence of HPCD-PSC/PTX nanoparticles. A pharmacokinetic study was determined on three parameters of PTX and results obtained were as follows: (area under curve (AUC): 2.57 μg⋅h/mL, body clearance (CL): 1.75 L/h/kg), plasma elimination half-life ((t1/2): 0.45 h, PS (AUC: 3.31 μg⋅h/mL, 1.35 L/h/kg and 0.66 h)) and PSC (5.07 ± 0.68 μg⋅h/mL, 1.03 ± 0.17 h, 0.99 ± 0.13 L/h/kg), respectively. It was thus concluded that PSC has a 2-fold increase in the AUC and 2-3 fold increase in t^1/2^ compared to the PTX; therefore, PSC could expect a sustained and prolonged release of PTX to cancer cells. On the basis of these results, PSC was found to be the most assuring delivery vehicle for breast cancer with lesser renal toxicity [21].

While much has been said about PTX, curcumin is another important chemosensitizer agent that has been used in combination with PTX against several types of cancer. Baek et al. prepared PTX- and curcumin-loaded lipid NPs (PCN), PTX-loaded SLNs and folate-conjugated curcumin (FPCN). An in-vitro study of drug release for FPCHN and FPCN assisted PBS with different amounts of HPCD (0, 10, 30, or 50 mg) that were added to form an IC with curcumin. In the first step release of the CUR from FPCHN-(10, 30, and 50) at 2 h, it was found to be 32.3 ± 4.7%, 41.3 ± 6.8%, and 63.8 ± 7.1%, respectively. All CUR were fully released in 12 h, which is faster than PTX. FPCHN-30 was selected for further studies. After 8 h treatment of curcumin with PTX, higher absorption of PTX, along with the CUR in MCF-7/ADR (5.1 ± 0.3 ng/μg), than free PTX (1.1 ± 0.1 ng/μg) was demonstrated. FPCHN-30 exhibited the most PTX uptake (19.1 ± 1.1 ng/μg) and it was 1.44 times greater than FPCN. All of the NPs presented time-based cytotoxicity. From the above result, it was concluded that cellular absorption of PTX/CUR into MCF-7/ADR was enhanced in the presence of folate receptors and p-gp and the FPCHN-30 nanocarrier shows great potential to suppress MDR tumor cells [22].

The hydrogel-mediated dual drug delivery process was studied by Hyun et al. Based on the drug DOX and HCl, PTX DOX·HCl/PTX-complex β-CD-loaded GC hydrogel (GDCP), Glycol chitosan (GC), and (β-CD) were used as an excipient to dissolve PTX in water. An in-vivo and in-vitro study was conducted for breast cancer using MCF-7 cells to understand the therapeutic effects. In the case of in-vivo, the anticancer effect of GDCP was found to be excellent, as compared to the control and DP-treated tumors. The anticancer impact of GDCP in mice was investigated by injecting MCF-7 cells. Gross appearance of the tumor was tested with GDCP, DP, and the control for 1, 3, 5, and 7 days. In the control, from 0 to 7 days, the size increased from 173 mm^3^ to 356 mm^3^, while DP and GDCP exhibited a gradually decreased size of the tumor (176 mm^3^ to 131 mm^3^ and 178 mm^3^ to 60 mm^3^). GDCP showed a greater anti-cancer effect than DP. Therefore, GDCP can, thus, be considered as a potential clinical therapy for breast cancer [23].

Sharker et al. developed a cooperative DDS to detect and target particular cancer sites (internally and externally) with controlled drug release. The system has an environment-sensitive pH. It also has a photo-thermal, remote-controlled drug delivery matrix, which can be controlled externally. The heat produced photothermally provides an optical signal. In the present study, they used partially carbonized fluorescence hyaluronic acid (HA-FCN) created by the carbonization of HA combined with boronic acid (BA) to form β-CD (HA-FCN-CD) and the PTX hydrophobic drug utilized for targeting cells. The characterization of active TDS of HA-FCN-CD-paclitaxel was performed to determine their shape and size and the TEM results revealed that nano-carriers comprised of spherical particles 70 ± 6 nm in size. The atomic force microscopy measurements included a 75 nm diameter with 8–9 nm identical thickness. PTX was released efficiently from the IC drug carrier to suppress the cancer cells and controlled the burst release that occurred after the photothermal (NIR) heat application. It was concluded that this system has the power of target control along with real-time bio-imaging and shows the considerable effect on improving drug efficacy [24].

**Figure 2:**
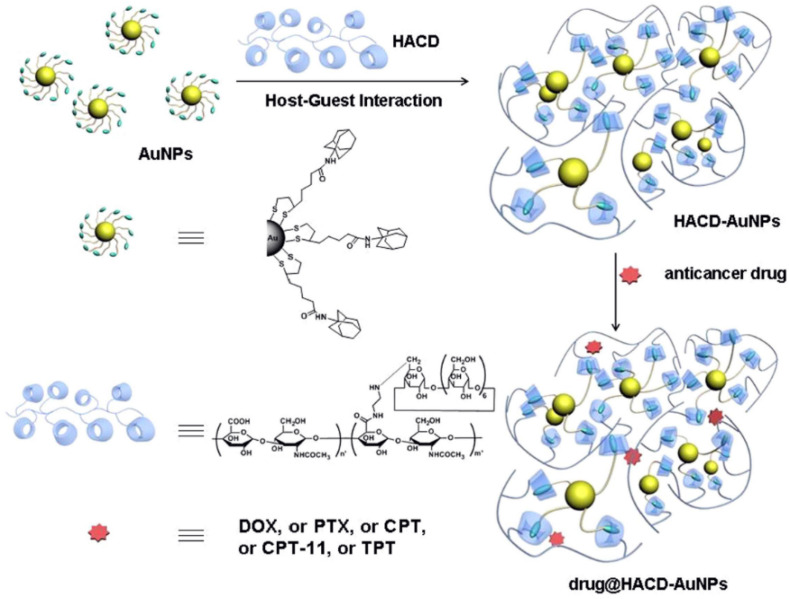
Schematic illustration of the chemical structures and construction of the HACD-AuNPs and the drug @HACD-AuNPs. (Adapted with permission from Ref. [25]).

Li et al. constructed supramolecular nano-assemblies using l polysaccharide-gold nanoclusters (HACD-AuNPs) conjugated with gold nanoparticles (AuNPs) and cyclodextrin implanted with hyaluronic acid (HACD). These conjugates are biocompatible, as well as versatile, for loading and delivering different anticancer drugs, such as PTX, topotecan hydrochloride (TPT), irinotecan hydrochloride (CPT-11), CPT (camptothecin), DOX and hyaluronic acid (HA) have the ability to identify metastatic tumors, as observed in Figure 2. Gold nanoparticles conjugated with HA can treat the HA degrading diseases. The inclusion ability of β-CD, including drugs such as DOX, CPT and PTX, was found to be 7.5%, 15.8% and 21.0%, whereas HACD AuNPs improved the efficiency of the drugs, which was observed to be 79%, 47% and 53%, respectively. Compared to β-CD, HACD AuNP nanoclusters has better entrapment efficiency. The zeta potential and the average diameter of HACD AuNP nanoclusters were found to be −30.99 mV and 258 nm, with a negative charge. EE (%) and LE (%) were observed for DOX/HACD-AuNPs (79, 11), PTX/HACD-AuNPs (53, 19), CPT/HACD-AuNPs (47, 5) CPT-11/HACD-AuNPs (45, 7) and TPT/HACD-AuNPs (34%, 5%), respectively. The DOX-HACD-AuNP’s formulation exhibited tumor inhibition abilities but showed lower toxicity due to the presence of the HA receptor than the free DOX. The above-mentioned result shows that the HACD-AuNP’s formulation is a very promising TDDS in cancer treatment [25].

Yan et al. synthesized HP-β-CD conjugates with biotin and arginine to form biotin-Arg-HP-β-CD complexes by coupling them with hydroxyl and carboxyl groups of biotin and arginine, which were used as functional spacers. PTX-loaded NPs were developed by a solvent evaporation technique. Characterization of the complexes shows that the zeta potential was −57.7 mV and diameter was 121.9 nm. Further in-vitro and in-vivo studies were performed to check the antitumor activity against the MCF-7 cell lines. After incubation for 3 h at 37 °C, the arginine-loaded nanoparticles increased the intensity of the fluorescence by 1.5 times against both complexes, which entered into the cell via endocytosis with the help of biotin receptors. The addition of biotin and arginine can enhance cellular uptake, while releasing more PTX into the targeted area. Further antitumor efficacy was examined using U14 mice with tumors using PTX/biotin-Arg-HP-β-CD NPs, which accumulated inside the tumor by the EPR effect and endocytosis. High cell penetration is enhanced by arginine. All these effects increase the concentration of PTX in tumors and showed excellent potential. PTX/ biotin-Arg-HP-β-CD NPs exhibit the significant ability of a tumor TDDS [26].

Earlier studies have reported several side effects, which occur due to the use of mixture of Cremophor and ethanol, which led to non-linear pharmacokinetics and PTX distribution. To overcome the problem associated with the solution Cremophor, the alternative technique used by Bilensoy et al. demonstrated a comparison for its safety and efficacy by using amphiphilic CD nanoparticles. PTX-loaded CD NPs (6-o-CAPRO-β-CD NPs) were formulated using the nano-precipitation technique to form nano-capsules (500 nm) and nanospheres (150 nm). Furthermore, encapsulation with the active ingredient PTX was carried out using two different approaches. Highly loaded nanospheres were prepared by preformed IC of PCX/6-o-CAPRO-β-CD with the molar ratio 1:1. In the next approach, conventionally loaded NPs were synthesized using a drug solution. The cytotoxicity study was performed using these NPs against L929 cells for their hemolysis using MTT assay. The results found a considerable variation in cytotoxicity of NP. PTX-loaded NP’s anticancer efficacy was measured against PTX in the Cremophor vehicle for MCF-7 cells. Amphiphilic CD NPs are found to be more effective than the combination of Cremophor and ethanol. Hence, the amphiphilic CD NPs are supposed to be the most considerable alternative formulation to administer PTX [27].

Varan et al. demonstrated PTX-loaded CD NPs using the nanoprecipitation technique and characterized them. The diameter was found to be 80–125 nm and the smallest diameter obtained NPs with polycationic (PC) β-CDC6. NPs with (+) charge improved PTX loading capacity up to 60% and maintained it for 30 days in aqueous solution. An in-vitro study showed that all blank CD NPs were nontoxic to L929 cell lines, whereas PTX-loaded NPs showed considerable anticancer activity on MCF-7 cell lines. The results show that amphiphilic CD NPs of different surface charges were found to be an effective and nanometer-sized drug delivery system for chemotherapy [28].

A similar complex was prepared by Bilensoy et al., where an inclusion complex was formed using PTX with amphiphilic CD, 6-o-CAPRO-β–CD.FTIR analysis depicted stretching vibrations for O-H, aliphatic C-H and C-O at 3700–3100 cm^−1^, 3000– 2850 cm^−1^ and 1800–1700 cm^−1^, respectively. Further studies revealed needle like crystalline structure when studied under SEM and NMR studies confirmed the shift in H-3 and H-5 by 0.02 and 0,03 ppm for 1:1 and 1:2 molar ratio complexes, respectively thus confirming formation of IC between PTX and 6-o-CAPRO-β–CD

IC prepared nanospheres and nano-capsules through the precipitation technique with nanosphere sizes of 150–250 nm and nano-capsules of 50–500 nm with zeta potential in a range of −18 to −39 mV. The in vitro release profiles showed nanosphere 12 h and nano-capsule 24 h prolonged release. Thus, it was concluded that amphiphilic NPs may provide alternatives for non-surfactant, Cremophor-free carriers for injectable use of PTX [29].

**Figure 3:**
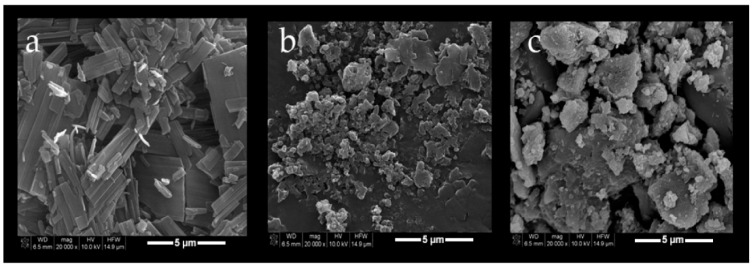
Scanning electron microscopy (SEM) photomicrographs of PCX (**a**); PCX:6OCaproβCD inclusion complex (**b**) and PCX:PC βCDC6 inclusion complex (**c**). (Adapted with permission from Ref. [30]).

In another study, amphiphilic CD NPs were found to protect PTX re-crystallization for aqueous distributions. Varan et al. developed PTX as a model anticancer drug with amphiphilic CD NPs for various surface charges using the freeze-drying method. Two amphiphilic non-ionic (NI) CD derivatives, heptakis (6-o-hexanoyl) and cyclomaltoheptose (6-O-CaproβCD), and a polycationic amphiphilic CD (PC βCDC6) were studied. The PTX-loaded nanoparticle drug stock solutions were prepared in ethanol and amphiphilic CDs were dissolved in PTX stock solution containing ethanol. The organic phase was converted to the aqueous phase using ultrapure water under a magnetic stirrer. The organic phase was then evaporated and NP dispersions were filtered for further study. The characterization study conducted on a PTX: CD inclusion complex and blank nanoparticles revealed the following results. Blank 6OCaproβCD, blank PC βCDC6, PTX/6OCaproβC, PTX/ PC βCDC6, PTX/6OCaproβC IC and PTX/ PC βCDC6 IC sizes were found to be in the range of 103 ± 1 nm, 75 ± 2 nm, 113 ± 4 nm, 82 ± 2 nm, 135 ± 2 nm, 120 ± 4 nm, the polydispersity index (PDI) was reported in the range of 0.13 ± 0.02, 0.16 ± 0.002, 0.22 ± 1, 0.24 ± 5, 013 ± 0.04, 0.15 ± 0.02 and the zeta potential was observed in the range of −24 ± 0.3, +61 ± 1.4, −29 ± 2, +62 ± 1, −31 ± 3, +59 ± 2, respectively. SEM analysis of blank PTX, PTX/6OCaproβC IC and PTX/PC βCDC6 IC confirm the inclusion of PTX within the cyclodextrin cavity, as observed in Figure 3. All nanoparticles show diameters ranging up to 150 nm. The result of the electrostatic interaction PC βCDC6 NPs showed high efficacy, almost 1.2 times higher than the negatively charged 6OCaproβCD NPs. Further cell culture study conducted using different cell lines and IC_50_ for PTX was set on 211.3 ± 4.3 nM for MCF-7 and 292.1 ± 5.2 nM for HDF cells lines. The 3D tumor model was used to observe the absorption and perforation of polycationic and NI CD NPs [30].

In another example, Baek et al. synthesized solid lipid nanoparticles (SLNs), of submicron-size (50–1000 nm). In the present work, PTX-loaded SLNs with hydroxypropyl HP-β-cyclodextrin (PSC) and without HP-β-cyclodextrin (PS) using hot-melted sonication were studied to examine the stability of PTX-loaded SLNs (PS) and (PSC). In-vivo and in-vitro analyses were carried out using MCF-7/ADR. The freshly prepared (PS) and (PSC) results demonstrated particle sizes of 294.4 ± 3.6 and 246.6 ± 2.8 nm, EE (%) of 71.1 ± 1.8 and 78.2 ± 7.1 % for (PS) and (PSC), respectively, while after 60 to 180 days (at respective temperatures), the size, PDI (polydispersity index) and EE of both the NPs differed significantly. Similarly, the in-vitro release profile of PTX from PS and PSC shows the release of PTX (43.2 ± 8.5% and 32.6 ± 3.4%, respectively) within 2 h and the zeta potential was observed to be −37.9 ± 2.5 and −51.4 ± 5.5 mV. All the above results demonstrated that the size of PSC was < 300 nm and for PDI, it was 0.3, which can be considered for oral delivery. PS showed a change in cytotoxicity and increased the incubation time, whereas PSC exhibited no change for storage at 40 ± 2 °C, and was more stable than PS and PDI. Thus, the results indicated that PSC was found to be a better formulation for enhancing the stability of drugs [31].

### 2.2. Cyclodextrin Nanoparticles Used for Lung Cancer Therapy

Lung cancer is ranked the highest for males and second among females with regard to cancer deaths. Toxicity in chemotherapeutics and MDR are major hindrances in successful chemotherapy. A safe and innovative sensitizer is required to solve the MDR problem. Shen et al. developed a novel biodegradable PTX/hydroxypropyl-β-CD complex loaded with liposomes (PTXCDL) as a nano vehicle to encapsulate the hydrophobic drug in the internal water phase. Therefore, the drug/CD inclusion complex was inserted into the hydrophilic inner core, which improved the loading efficiency, bioavailability and poor solubility of the drug. In the present study, PTXCDL (TEM and DLS: 80–90 nm size) was developed for treating the PTX-resistant A549/T cell-line. The in vitro studies show that PTX release is dependent on pH-sensitivity, increases the intracellular uptake and potent cytotoxicity where HP β-CD prohibit the marginal discharge of the PTX-resistant cancer cell. The in-vivo study showed that the antitumor efficacy significantly improved due to the presence of the PTXCDL group. The apoptosis rate for PTXCDL was found to be 29.6% higher than the PTXL; thus, it suggests that PTXCDL is effective in delivering drugs with low water solubility by enhancing their therapeutic effect in treating MDR cancers using PTXCDL [32].

Yuan et al. prepared a nano assembled drug delivery system using the inclusion complex of PTX with β-CD with poly-acrylic-acid (PAA) (P-CD-PAA) to develop P-CD-PAA-PTX NPs. It demonstrated improved aqueous solubility from 0.34 to 36.02 µg mL^−1^ for PTX. A double hydrophilic polymer made up of PAA and side-chains of β-CD constructed a comb-like polymer. The PAA backbone provided negative surface charges that gave stability and prolonged the physiological condition. The formation of P-CD-PAA-PTX NPs was performed by dissolving PTX into ethanol to prepare the stock solution and 1 mL of PTX: ethanol solution was added to the 1 mL of PCDAA solution and the mixture was stirred for 3 days. Ethanol was removed by the vacuum evaporation technique and the resultant solution was then filtered using a 0.22µm cellulose acetate filter membrane and was further lyophilized. The size of the NPs was measured by TEM and DLS, which showed a spherical shape and size of 100–200 nm, with a size distribution of 170 ± 12.4 nm. Further in-vitro study using Taxol and with the formulation was conducted against the HeLa cell line by incubating samples at regular time intervals was of 12 h, 24 h and 48 h. Cell viability was observed against lowering incubation time. The formulation showed cytotoxicity against cancer cells. Characterization was performed by near-infrared fluorescence imaging (NIRF), which showed bio-distribution using NIRF dye with NIR-797 that was injected into H22 mice with tumors. Signals for NIFR were found within the intestine and liver after 3 h. The antitumor effect studied with H22 had an inhibition rate of 44.76% and their survival rates with saline, Taxol, P-CD-PAA polymers and P-CD-PAA-PTX NPS were recorded and noticed that within 41 days, 6 of 10 mice died. Thus, it was indicated that PTX-P-CD-PAA NPs have an excellent antitumor effects and application in PTX drug delivery [15].

Tripalmitin (glyceryl tripalmitate solid lipid NPs) complexed with PTX (tripalm-NPs-PTX) and with hexaethylene glycol, macelign and β-CD was developed by Leiva et al. All the NP-PTX formulations presented excellent hemocompatibility and significant antitumor effects in different cancer cell lines (MCF-7, MDAMB231, SKBR3, T47D, A549, NCl-H520, and NCl-H460). The IC_50_ of tripalm-NPs-PTX was 40.5 times lower in breast cancer and 38.8 times for lung cancer. Similarly, the IC_50_ of cancer stem cells for lung and breast cancer cells decreased significantly (6.7 times for MCF7 and 14.9 times for A549). Hence, it was concluded that the new nano-formulation of PTX can improve the antitumor activity and act as a novel DDS for treating lung and breast cancer [33].

The gold nanoparticles (GNPs) showed excellent characteristics, including robust stability, controlled size, biocompatibility, and tunable optical properties. In the case of MDR (multi-drug resistance), GNP-loaded anticancer drugs show great potential, increasing efficacy in PTX-resistant cell lines. Based on this progress, supramolecular conjugates successfully constructed from AuNPs (GNPs) with β-CD enclosed PEG molecules and PTX molecules and prevented the over-expression of Pgp proteins in drug-sensitive cells. The cytotoxic study conducted using the NSCLC cell line H460, incubated with PTX or PGNPs, showed that it increased from 10 to 100 nM for 100 days. The resulting H460 sub-lines of H460_PGNP_ cells were found to be 28.8 nM (increased Pgp expression level). The H460 PTX could not increase the Pgp expression level. The E_50_ value for H460 PTX was 346.3 nM, which is 54 times the regular H460. It suggests that PTX has more resistance. It can be concluded that nano drug conjugates can prevent drug resistance and ignore Pgp-treated drug resistance. It has better cytotoxicity for cancer cells with drug resistance [34].

### 2.3. Cyclodextrin Nanoparticles Used for Ovary Cancer Therapy

Ovarian cancer is the most fatal gynecological cancer. Chemotherapeutic treatment is harmful to healthy cells, shows unfavorable biodistribution and causes side effects, such as neuropathy, hair loss, bone marrow suppression, cardiotoxicity, nausea etc. [35]. Sharma et al. disclosed that the variety of different CDs (HP-β-CD, DM-β–CD, HE-β-CD) can increase the solubility of PTX 2 × 10^3^ fold or more, without changing the cytostatic properties. They studied the biological effect using Hey-1b and obtained the result of 50% inhibition of cell growth with the 5–10 nM drug. In addition, spectroscopic (NMR, IR, and circular dichroism) and thermal study suggested that the formed inclusion complex is weak in solution but stable in the solid-state. Mixed ether-ester derivatives of β–CD can reduce the quantity of CD-mediated toxicity. It can achieve marginal safety that is acceptable for the dose-limiting toxicity [36].

In the present experiment, Boztas et al. used paclitaxel and curcumin with a new carrier, poly-(β-cyclodextrin-triazine) (PCDT), to form a novel drug delivery system through one-step condensation polymerization. The phase solubility analysis of Cur and PTX was improved significantly with PCDT encapsulation. The Cur complexation 16% (*w*/*v*) concentration of PCDT improved solubility up to 35.5 mg/L, i.e., 3500 times more compared to the initial value. Similarly, for PTX with PCDT 12% (*w*/*v*), it was found to be 941 times. Cell viability studies were conducted using MTT assay for A2780 and SKOV-3, DU-145, H1299 and MCF-7. IC_50_ values of PTX/PCDT and Cur/PCDT on MCF-7, DU-145, SKOV-3, A2780 and H 1299 were found to be as follows: A2780, 1.2a ± 0.7 + 5.0; SKOV-3, 2.1a ± 1.3 + 5.0; H1299, 10.3a ± 3.9 + 5.0; DU-145: 4.3 ± 1.0 + 5.0 and MCF-7: 3.8 ± 1.4 + 5.0, respectively. The MTT assay suggested that compared to free PTX and curcumin, the complexed Cur/PCDT, depicted 30% lower IC_50_ value, thus indicating that the complexed structure inhibited the cell propagation in all five cell-lines. Finally, they evaluated the synergistic interaction between the PTX and Cur combination index (CI) against all five cancer cell lines. The results indicated that a major improvement in the effectiveness of the CUR/PCDT complex in all cell-lines against the free CUR. PTX/PCDT complex is more efficient with regard to remediation, as compared to free PTX. Hence, it is concluded that PCDT is important in hydrophobic drug delivery. A synergistic interaction was observed between Cur and PTX with PCDT for various carcinoma cells. Therefore, encapsulation of hydrophobic drugs with PCDT is beneficial in treating lung and ovarian cancer [37].

Bhatt et al. used the combination strategy to create an inclusion complex to improve loading capacity. Two complexes were made using 2,6-di-omethylbetacyclodextrin (DMβCD) and double-loaded PEGylated liposomes (DLPLs) conjugated with PTX by the thin-film hydration method. A characterization study was performed with DSC, XRD, FTIR for PTX, DMβCD, PTX/DMβCD (IC) and it was observed that the melting point for PTX was around 218 °C. The DSC spectrum of PTX/DMβCD IC confirmed the amorphization of PTX. XRD showed PTX’s crystalline nature, whereas DMβCD was amorphous. IC displayed the diffused pattern at the 10–15° 2θ area, which confirmed the presence of PTX in an amorphous state upon complexation. FTIR analysis confirmed the complexation between PTX and DMβCD. It was observed that the spectra for the PTX drug showed prominent absorption bands from 1730 cm^−1^ to 1600 cm^−1^, 1380 cm^−1^ to 1180 cm^−1^, and 900 cm^−1^ to 710 cm^−1^. Similarly, stretching of the atoms for DMβCD was found at 3430 cm^−1^ to 3420 cm^−1^. After complexation of the IC, a PTX molecule stretching carbonyl ester band was found at 1724 cm^−1^, which enhances the intensity and their lyophilized form was observed to be stable at 2–8 °C for 90 days. TEM images showed that DLPL particle size was around 130 nm. Furthermore, a cytotoxicity study was performed by MTT assay using SKOV3, ovarian cancer cell-lines, and the IC_50_ value increases by 4.2-fold in 48 h. An in vivo study shows an increase in the circulation time and concentration of the plasma in DLPL, in comparison to Taxol. Hence, the formulated complex shows great potential for the currently marketed PTX formulation [38].

### 2.4. Cyclodextrin Nanoparticles Used for Cervical Cancer Therapy

Cervical cancer is the leading cancer among females. Vaginal drug delivery (VDD) is considered to be a favored alternative for treating cervical cancer. A nanogel with a multivalent interaction delivery vehicle was proposed by Qian et al., which has the potential to serve as an efficient delivery system for cervical cancer treatment and multi-drug-resistance (MDR). Poly (acrylic acid) (PAA) was selected as the backbone of the hydrogel that can tighten the junction between epithelial cells that promotes drug release in mucus. The chemotherapeutic drug PTX uses (β-CD) to enhance solubility. Various inclusions were formed using β-CD with PTX. The nanogel was created using PAA-β-CD and PAA-TAX, which were used for examination. The uptake behavior of the nanogel was verified using HeLa cells tagged with Nile red. The results concluded that it increased Nile red intensity and the nanogel was internalized effectively inside the cells. The results of MDR and free TAX were measured. The proportion of apoptosis was found to be 58.2% for MDR of the PAA- β-CD/PAA-TAX nanogel, which exhibited more cytotoxicity than free TAX (56.0%). The resistance index (RI) values for TAX and PAA-β-CD/PAA-TAX were calculated as 54.44 ± 4.11 and 2.86 ± 0.01, respectively. The nanogel improved the solubility, enhanced the stability and protected leakage before target site delivery of the hydrophobic drugs and it was concluded that it exhibited outstanding resistance to MDR and inhibited cervical cancer significantly [39].

**Figure 4:**
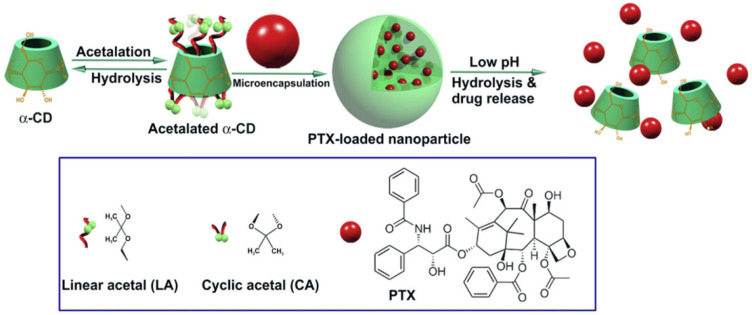
Schematic illustration of the construction of pH-sensitive PTX nanoformulation based on acetylated a-CD (Ac-aCD). (Adapted with permission from Ref. [40]. Copyright © 2013 Elsevier Ltd).

The functionality of nano-carriers is depicted by their success in preclinical and clinical effects on treating tumors. He et al. prepared a pH-sensitive nano-system against multidrug-resistant cancer cells. It was prepared by synthesizing acetylated α-CD (Ac-aCDs) nanoparticles using a simple solvent evaporation technique with controlled sizes and distribution, as presented in Figure 4. A cell culture study was performed for cell lines MDA-MB-231, HeLa, B16F10 and HepG2 using Ac-aCDs 180 and Ac-aCDs 480 NPs. A high concentration of NPs lowers the cell viability and cytotoxicity of various NPs. An in-vitro study was performed for both resistant and sensitive cancer cells with MTT assay. For 24 and 48 h of incubation, the IC_50_ values were found to be 44.3, 70.7, 39.7, 47.6, 23.0 and 39.8 µg/mL and 37.7, 53.2, 33.7, 24.6, 20.5 and 31.2 mg/mL for PTX, PLGA NPs, Ac-aCD 10 NPs, Ac-aCD 90 NPs, Ac-aCD 180 NPs and Ac-aCD 480 NPs, respectively. The effect of the PTX nano formulation was further examined against MDR MDA-MB-231 and MCF-7. The IC_50_ values of PTX, PLGA NPs, and Ac-aCD180 NPs were found to be 115.8, 51.0 and 10.5 mg/mL and 14.1, 9.3 and 8.4 mg/mL. The results demonstrated that for the cytotoxicity study using PTX/Ac-aCD 180 NPs, was sensitive as well as resistant carcinomas were observed. The in-vivo study performed examinations of the antitumor activity using mice with melanoma. Mice were implanted with a fragment of the B16F10 tumor and inoculated with PTX/ Ac-aCD NPs at lower, medium, and higher doses. After 7 days, a single dose of 10 mg/kg hindered the growth of the tumor. Both the in-vivo and in-vitro study showed the loading capacity of PTX using pH-triggered acetylated α-CD nanoparticles, which have a more powerful drug loading capability, they enhance antitumor activity and reduce side effects [40].

Namgung et al. developed a nano-assembly of a multivalent targeted drug delivery system using a freeze-drying method for the polymeric cyclodextrin (pCD) and paclitaxel (pPTX) complexes. The nano assembly formation was investigated by intracellular esterase. The TEM characterization shows small ellipsoidal particles around 50 nm. The dissolution of PTX increased in the pPTX/pCD nanoassembly by the water-soluble carboxylic group present on the backbone of the polymer. The steadiness obtained was higher in the multivalent inclusion complexes (IC) pPTX/pCD (10^4^ times K_a_ value^)^ than the single PTX/CD complex. MTT assay was performed to study the therapeutic effects against HeLa, MCF-7, and HCT-8. The IC_50_ values were found to be 6.62 ngmL^−1^, 0.08 µgmlL^−1^ and 7.84 ngmL^−1^, respectively. Antitumor activity was examined with a HCT-8 xenograft mouse model against Taxol, pPTX, pPTX/pCD, CD, and saline. Notable results were observed for the pPTX/pCD nanoassembly, which decreased the tumor significantly and increased the survival rate [41].

Another mixture consisting of an anticancer drug (PTX) and antiviral cidofovir (CDV) was produced by the inkjet printing technique using adhesive film to treat cervical carcinoma. Varan et al. used PTX/HP-β-CD IC and NPs with PEG-PCL loaded on the CDV drug. For better solubility and release of the drug cidofovir, it was encapsulated withinpolycarprolactone NPs. An in vitro and cell culture study showed that the present formulation is more effective on human cervical cancer. The study proved that the inkjet printing technique has future potential for the development of antiviral and anticancer drugs [42].

Yin et al. prepared supramolecular hydrogel-based nanoparticles by using α-cyclodextrin (α-CD) and PTX conjugated mPECT (methoxy poly ethylene glycol-b-poly-ε-caprolactone-co-1,4,8-trioxa (4.6) spiro-9-un-decanone (PTX/mPECT NPs) to form IC. PTX-conjugated mPECT NPs were synthesized using the nanoprecipitation method. The synthesized NPs were then added to 0.2 mL aqueous solution of α-CD and stirred. After a few seconds, the gelation of PTX/mPECT NP/α-CD^gel^ was observed for sustained drug delivery, which was lyophilized and freeze dried. As the rheological study shows, PTX/mPECT NPs/α-CD is more injectable. The in-vitro study demonstrates the sustained and increased release of the drug of nearly 35% after a period of 20 days. A cytotoxicity study using MTT assay was performed on HeLa and 7703 cells. The results obtained from the methods demonstrated that these nanoparticles efficiently delivered and destroyed the cancer cells and suppressed the tumor growth. Hence, this newly formed PTX/mPECT NP/ α-CD delivery system could be a superior technique that reduces the side effects via local administration [43].

### 2.5. Cyclodextrin Nanoparticles Used for Liver Cancer Therapy

Cancers of the liver are the fifth and eighth most frequent cancer in males and females, respectively [44]. Previously, liver cancer was mainly treated by chemotherapeutic agents, apart from surgery. Most of the anticancer drugs have high toxic effects that cause severe side effects. Nowadays, polymeric NPs are utilized as effective targeted DDS fo improved curative effects with minimal impacts on healthy organs [45].

Amphiphilic CD aggregates immediately with a functional host site and with significant prospects in DDS. Sun et al. synthesized β-CD modified with an anthraquinone moiety and this was characterized using TEM (spherical 200 nm), SEM (bilayer length 4 nm), EFM, and DLS (hydrodynamic radius of 135 nm and diameter 270 nm), respectively. The formed vesicle was then studied using molecular dynamics (MD) or simulation and the formation mechanism as per 2D ROESY. The UV visible result showed that the anthraquinone moiety was clustered via its π- π stacking nature, as confirmed by the MD simulation and confined the CD cavity in the vesicle using 2D NMR ROSEY. The relation between PTX and H-4/H-2 of the CD moiety was confirmed. The outer cavity proton showed that PTX was located inside the hydrophobic region of 1/PTX. The incorporation of PTX decreased the hydrophilicity of amphiphile, which formed a loose 1/PTX vesicle structure that can be sustained for a month at 20 °C. The secondary loading that belonged to the CD moiety was ferrocene (FC) (1/FC) in the exclusion complex vesicle model. The 1/PTX vesicle was treated with 1/FC and characterized using TEM and SEM. The 1/PTX/FC vesicle had a smaller diameter than 1/PTX and the yield obtained for the complexes 1/FC and 1/PTX/FC Δδ using 2D NMR were as follows: H-1, −0.001; H-2, −0.001; H- 3, −0.002; H-4, −0.002; H-5, −0.002; H-6, −0.001, respectively. The anticancer drug PTX failed to replace FC in the CD cavity, which tends to enter the anthraquinone moieties. Further, cytotoxicity study revealed that the 1/PTX complex is superior to the natural PTX in the treatment of various carcinomas [13].

With regard to the PTX and β-CD interaction dimer, Pei et al. developed a stable supramolecular binary vesicle. CD/C6 IC was prepared using the nanoprecipitation method, which exhibited nano-aggregates in the solution. The DLS result showed the narrow distribution of hydrodynamic size 230 nm, the height image, and AFM and the width of the assemblies that was found to be 250 nm and 25–40 nm in height results in oblate and flattened assemblies on the silica disk surface. For the anticancer efficiency test performed using MTT assay, the CD with HepG2 incubation showed rapid activity, and groups that contain PTX, such as C6 NPs, Taxol, CD/C6 NVs and CD/NP(C6) NVs, show great potential in cancer cell killing. The results indicated that the PTX dimer showed an efficient therapeutic effect once it entered the tumor cells [5].

Cheng et al. developed the star shaped copolymer β-CD/g/(PNIPAAm-b-POEGA) poly-oligo-ethylene glycol-acrylate-(POEGA,poly-N-isopropylacrylamide-PNIPAAm) through host–guest interactions by forming inclusion complexes. This supramolecular nano-assembly promoted enhanced cellular intake of chemotherapeutics. The MDR-1 cells showed greater therapeutic effectiveness for conquering MDR tumor growth in the mice model. They established a xenograft nude mice model and PTX was then delivered in the form of β-CD co-polymer IC. The encapsulated drug significantly inhibited the HepG2 and MDR1 tumor growth (157 ± 68 mm^3^), compared to the saline treatment and Taxol, which were found to be 73 ± 17 mm^3^ to 1,552 ± 584 mm^3^; 1,242 ±727 mm^3^, respectively. The output from the above study indicates the presence of the nanocarrier system, which has great potential in drug-resistant tumor therapy [46].

### 2.6. Cyclodextrin Nanoparticles Used for Prostate Cancer Therapy

Prostate cancer is the most frequent and most fatal male cancer observed in the US. Therapies such as adjuvant hormonal therapy, radiation and surgery can cure cancer in its early stages [47]. Therefore, a delivery system that has great potential in cancer treatment needs to be developed as soon as possible. To solve the problem related to hydrophobic anticancer drugs, the β-cyclodextrin-poly (N-isopropyl acrylamide) (β-CD-(PNIPAAm)) star polymer nanocarrier was synthesized by Song et al. This polymer-encapsulated PTX improved water solubility and thermo-responsive PTX delivery to cancer cells by forming host–guest interactions at room temperature. Characterization of IC by NMR, FTIR, DLS and an in-vitro cytotoxicity study were conducted against MDR negativeAT3B-1-N and MDR +positive AT3B-1-N cells. The results concluded that a nanocarrier with thermo-responsive properties demonstrates better cytotoxicity at 37 °C than at 25 °C with improved solubilization of PTX in aqueous solution. The therapeutic effect and cellular uptake results overcame the MDR effect in cancer-infected cells, thus confirming potential treatment against cancer cells [12].

Alcaro et al. prepared an IC of PTX with various CDs, namely, 2,3,6-trimethyl-β-CD, 2,6-dimethyl-β-CD and β-CD using the freeze-dryer technique. It led to the formation of an amorphous sample, which was used to understand the formation of the PTX–CYD complex. Characterization using FTIR showed the carbonyl-ester band for PTX at 1730 cm^−1^ and broadening of the carbonyl stretching was found at 1600–1650 cm^−1^. The DSC results showed dispersal of the drug fusion, which peaked at 222 °C for the solid state. An additional study conducted using molecular modeling (using a three-step approach) investigated the interaction of PTX–CYD to clarify the energetic and structural recognition process. In the first step, they used the Monte Carlo (MC) Macro Model Package and selected the MM3 tool using the software MOLINE. In the second step, they adopted three open confirmations of CDs for comparing the results of docking. The third step involved a minimal energy complex (3 kcal/mol) for full energy refinement, applying different forces. Further energetic thermodynamic comparison experimental results were used to identify the most accurate computational protocol required for structural characterization. An in-vitro study was conducted using DU145 cell lines against free PTX and complexed PTX-(1, 2, 3), with the dilution factors of 1:50, 1:100, 1:500 and 1:1000, respectively. The results calculated as % cellular survival against the function of drug dilution complexes showed different antitumoral activities, but particularly PTX-1 showed greater antitumoral activity in comparison to the drug alone. The % cellular survival against the function of drug dilution for PTX-1 was observed as (1:50 12.2 ± 2.1, 88.9 ± 0.4, 1:100 22.3 ± 2.3, 90.6 ± 0.9, 1:500 23.1 ± 1.9 90.2 ± 0.5 and 1:1000 24.2± 2.6 91.5 ± 0.3). PTX-2 and PTX-3 transferred the drug from the CD cavity to the targeted cells. The researcher confirmed three conclusions based on the experiments, which as follows: 1. CYD has a hydroxyl group that controls the recognition of antitumor compounds. 2. Of all the complexes, PTX-2 demonstrates better stability. 3. The in vitro findings show that the solubility of PTX increases with the CYD complex [48].

### 2.7. Cyclodextrin Nanoparticles Used for Colon Cancer Therapy

Every year, 1.6 million patients are diagnosed with colon cancer. Conventional chemotherapy involves delivering drugs to non-targeted areas, which results in unwanted side effects, such as nausea, vomiting, diarrhea, etc. The treatments to date have not achieved satisfactory results. [49,50] Jeon et al. developed supramolecular NDDS by magnetic-iron-oxide NPs, β-CD, and polymerized PTX. The pPTX/CD-SPION were synthesized using the co-precipitation method by dissolving pPTX in 20% ethanol solution with PBS of pH 7.4 and then, the solution β-CD-SPION was dissolved in 20% ethanol solution, which was added into the pPTX solution drop by drop and stirred at room temperature. The resulting pPTX/CD-SPION solution was dialyzed using DI water and stored at room temperature. The size and zeta potential of the thermally cross-linked SPION nanoparticles were found to be −28.10 ± 0.26 mV and 221.07 ± 28.72 nm, respectively. An in vitro study against HeLa, MCF-7, and CT-26 indicated good biocompatibility and showed low cytotoxicity compared to free PTX and increased the accumulation of pPTX/CD-SPION NPs in the targeted cells magnetically. A hemolysis assay was performed using NPS and negligible hemolytic effects were found on the erythrocytes against PTX alone. The synthesized DDS is good for treatments and has potential with regard to its targeting ability [51,52,53,54,55,56]. The important findings and applications of cyclodextrins for treating several types of cancer are described in Table 2.

## 3. Conclusions

Different types of delivery systems, such as liposomes, polymeric nanoparticles, nano-capsules, nanospheres and micelles containing various types of cyclodextrins, can increase loading capacity, solubility and stabilize the chemotherapeutic drug paclitaxel to reduce its toxic effects. Several types of cancers, such as breast, lung, prostate, multi-drug resistant, ovarian, cervical cancer, etc., are treated using bio-degradable CD nanoparticles. They were studied as nano-carriers for delivering the drug across the cell membrane and reducing the off-target effect. Drug/CD inclusion complexes were pushed into the inner core of liposomes, which is hydrophilic and is considered to be a novel drug delivery vehicle. It improves the bioavailability, loading efficiency, and low solubility of the drug. CD-based nano assemblies offer effective cytotoxicity against various cancer cell lines. Polymeric cyclodextrin is an upgraded therapeutic vehicle compared to native cyclodextrin. The CD–PTX inclusion complex acts as an enhancer in anticancer therapy to evade issues related to solubility and stability, which are discussed in detail. PTX-loaded NPs show a prominent anticancer effect for the MCF-7 human breast cell line. According to the results of this study, amphiphilic CD NPs of different surface charges were found to be the most favorable alternative for self-assembled nanometer-sized DDS. In silico studies were performed using molecular docking and simulations for understanding aspects such as the binding energy, stability of the compound and host–guest interactions. One should perform an in silico study before proceeding to the practical application for better results. The conclusion of the study was that CD-PTX nano-formulations can substitute the present intravenous infusion of PTX and are able to stop the use of Cremophor EL.

Currently, clinical trials using CDs in combination with anticancer medications in the treatment of various types of cancers are being conducted. Unfortunately, the majority of the trials are currently in the recruiting stage and do not yet have any conclusions. However, it is crucial to watch these clinical trials, since it is useful to study CD’s applicability for cancer treatment and possible applications of new CD-based delivery methods in clinical practice may soon make use of novel CD-based delivery systems. New nano-formulations of PTX can improve antitumor activity and serve as an NDDS for the treatment of breast and lung cancers. In conclusion, this study critically evaluates CD-based delivery systems for anticancer therapy that have been tested in vivo and in vitro.

## Figures and Tables

**Table 1:** Physico-chemical characterization of PTX-loaded poly(anhydride) nanoparticles. Experimental conditions: poly(anhydride): 100 mg; paclitaxel: 10 mg; PTX–cyclodextrin stoichiometry: 1:1. Incubation time: 30 min. Data expressed as the mean ± SD (n = 6). EE: Encapsulation efficiency. Adapted with permission from [19]. Copyright © 2010 Elsevier.

	Size (nm)	Zeta potential (mV)	Yield (%)	PTX content (μg/mg NP)	EE (%)
NP	179 ± 2	−48.1 ± 0.8	91.3 ± 3.1	–	–
PTX-NP	204 ± 4	−38.3 ± 2.1	51.2 ± 6.6	0.29 ± 0.1	0.14
PTX-CD NP	298 ± 6	−39.3 ± 5.2	68.6 ± 4.4	38 ± 3.1	28.1
PTX-HPCD NP	307 ± 7	−42.1 ± 1.4	63.3 ± 2.9	167 ± 8.3	97.4
PTX-NHCD NP	310 ± 6	−34.5 ± 3.9	59.5 ± 4.6	91 ± 7.6	61.6

**Table 2:** Details of application of cyclodextrin paclitaxel nanoparticles, with a focus on type of nanoparticles, cyclodextrin type, preparation technique and type of cancer treated.

Types of Nanoparticles (NPs)	Types of Cyclodextrin	Types of Cancer/Cell Lines	Preparation Technique	Miscellaneous	References
C6 NPs, CD/C6 NVs and CD/NP (C6) NVs	β-cyclodextrins (β-CDs)	HepG2 cells -Liver cancer		^1^H NMR, ROESY, XRD, FTIR, TEM, SEM, AFM, DLS	[5]
FCD-1 and FCD-2 NPs	Amphiphilic CD derivatives	Breast cancer L929 cell, T-47D, and ZR-75-1 human breast cancer cells.	Freeze drying	Student’s *t*-test, F-test, ANOVA, SEM, ^1^H NMR, DSC,	[6]
FCD-1 and FCD-2 NPs	Amphiphilic CD	Breast cancer, 4T1 cancer cells, female Balb/c mice	Freeze drying	ANOVA, F-test	[7]
β-cyclodextrinpoly (N-isopropylacrylamide) star polymer	β-CD, Me-β-CD, HP-β-CD	AT3B-1 cells	Lyophilization Freeze drying	^1^H NMR, FTIR, DLS, DSC	[12]
mono [6-deoxy-N-ethylamino-(N′-1-anthraquinone)]- β-cyclodextrin nanostructure	β-CD	Liver cancer HCC cell line, HepG2		TEM, SEM, DLS, 2D NMR ROESY	[13]
Synthesis of P-CD-AA-PTX NPs	β-CD-modified poly (acrylic acid)	H22 cell line -Lung cancer	Lyophilization	DLS, TEM, FTIR, IR, ^1^H NMR	[15]
PTX-CD NP, PTX-HPCD NPs	β-CD, HPCD, NHCD	Metastatic breast cancer Male Wistar rat		Mann–Whitney U-test	[19]
PTX–CD complex	Hydroxypropyl β-cyclodextrin, methyl β-cyclodextrin	PC-3, A2780, MCF-7, HT-29 and A549 -Prostate cancer, ovarian carcinoma, breast cancer, colorectal adenocarcinoma, and lung cancer	Lyophilization	Statistical analysis: single-factor ANOVA, *t*-test H NMR, XRD	[17]
APPZ NPs	β-CD	BT474, SKBR3, OVCAR3, and HS5 cell lines -Breast cancer and ovarian cancer		FTIR	[18]
(HA-CD/PLL) capsules	β-CD, DM-β- CD, HACD	Breast cancer MDA-MB-231 cell line	Layer by layer deposition Host-guest complexation	^1^H NMR, SEM	[20]
PS and PSC SLNs	HPCD	Breast cancer MCF-7cells Female BALB/c nude mice	Hot-melted sonication	Student’s *t*-test	[21]
GDCP hydrogel	β-cyclodextrin	MCF-7 cells	Lyophilization	One-way analysis of variance (ANOVA)	[23]
HA-FCN (hyaluronic acid fluorescence carbon nanoparticles	β-CD	Breast cancer MDAMB-231 and MDCK (normal cells)	Freeze drying	NMR, TEM, XRD	[24]
Adamantylamine-modified gold nanoparticles (AuNPs)	HACD	Breast cancer, MCF-7, NIH3T3 (fibroblast cells)		XPS, HR-TEM, AFM, ICP	[25]
(biotin-arg(pbf)-HP-β-CD) NPs	HP-β-CD	Breast cancer MCF-7	Lyophilization	TEM, XRD	[26]
Nanosphere and nano-capsules of 6-0-CAPRO-β-CD	Amphiphilic β-CD, 6-0-CAPRO-β-CD	L929 mouse fibroblast cells, MCF-7 cell line, Breast cancer	Nanoprecipitation technique	SEM, AFM, two-way ANOVA, Kruskall–Wallis analysis, and Tukey test	[27]
6OCaproβCD, CS-6OcaproβCD, PC βCDC6	Amphiphilic cyclodextrin	Breast cancer MCF-7, L929 fibroblast cell line	Lyophilization	Student’s *t*-test	[28]
(6-0-CAPRO-β-CD) NPs	Amphiphilic cyclodextrin	Metastatic breast cancer	Lyophilization	FTIR, SEM, ^1^H NMR, DSC	[29]
PCX-loaded 6OCapro β-CD and PCX-loaded PC β-CDC6 NPs	(6OCapro β-CD) and (PC β- CDC6)	Breast cancer cells and fibroblast cells -Breast cancer	3D multicellular tumor model Lyophilization, freeze-drying method	FTIR, IR, SEM	[30]
PS-NPs, PSC-NPs, and SLN	Hydroxypropyl-β-cyclodextrin	MCF-7 ADR cells Breast cancer	Hot-melted sonication	Student’s *t*-test	[31]
paclitaxel/hydroxypropHydroxyl-β-cyclodextrin complex-loaded liposomes	HP-β-CD	Lung cancer, A549/T cells, Female Balb/c nude mice	Lyophilization	DSC, XRD, DLS, TEM	[32]
Tripalm-NPs	β-CD	Breast cancer and lung cancer -MCF-7, MDA-MB231, SKBR3, T47D - NCI-H460, A549, NCI-H520,		*t*-test ANOVA	[33]
GNPs, PGNPs	SH-β-CD	Lung cancer H460 and H460_PTX_ cells		TEM	[34]
PTX–CD complex	HP β-CD, DM β-CD, HE β-CD	Hey-1b, -Ovarian cancer -IC _50_: 5–10 nM drug	Rotary evaporation	NMR, IR Female Balb/C mice of ca	[36]
PCDT	β-cyclodextrin	Ovarian, lung, prostate, and breast cancer. -A2780, SKOV-3, H1299, DU-145, MCF-7	Freeze drying	^1^H NMR, FTIR, IC_50_ of curcumin-PCDT-7.7 and 13.4 μM	[37]
DLPLs	DMβCD	SKOV3 epithelial ovarian cancer cell line -Ovarian cancer -Sprague Dawley female rats -Female Balb/c mice	Modified co-solvent evaporation method Thin-film hydration method Lyophilization	DSC, FTIR, XRD, SEM, TEM Student’s *t*-test For liposome: 33 full factorial design	[38]
PAA -β-CD/PAA-TAX nanogel	β-CD	Cervical cancer HeLa cells and U14 cervical carcinoma cells expressing GFP (U14-GFP)	Esterification of PAA and TAX	^1^H NMR, FTIR, DLS, and TEM	[39]
Ac-aCD5 NPs, Ac-aCD15NPs, Ac-aCD 180NPs, Ac-aCD 240 NPs	α-cyclodextrin	B16F10, Hela, HepG2, MCF-7 and MDA-MB-231 cells, Female Balb/C mice Metastasis cancer, cervical cancer, liver cancer, and breast cancer	Solvent evaporation technique	DLS, FTIR, GPC, TEM, SEM	[40]
pPTX and pCD nanoassembly	Co-polymer: poly(isobutylrnr-alt-MAnh), poly [IB-alt-MAnh] and poly (methyl vinyl ether-alt-MAnh), poly [MVE-alt-MAnh]	MCF-7, HeLa and HCT-8 cell lines -Breast cancer, cervical cancer, and colon cancer	Multivalent polymer-polymer complex Freeze drying Molecular simulation of inclusion complex	DLS, TEM, H-NMR	[41]
CDV-PCL NPs	HP-β-CD	Cervical cancer L929 fibroblast cells, HeLa cells	Lyophilization	DSC, FTIR, SEM Student’s *t*-test	[42]
PTX-loaded mPECT NPs and PTX-mPECT NP/α-CDgel	Methoxy poly (ethylene glycol)-b-poly (ε-caprolactone-co-1,4,8-trioxa [4.6]spiro-9-un-decanone) (mPECT)	Human cervical cancer cells HeLa and human hepatoma cells 7703, Murine breast tumor 4T1, Balb/c mice Breast cancer and cervical cancer	Freeze drying	DLS, TEM, SEM, Student’s unpaired *t*-test	[43]
β-CD-*g*-(PNIPAAm-*b*-POEGA) *x* polymer	β-CD	HepG2 liver cancer/MDR1 cells or NIH-H460 lung cancer cells Liver cancer	Western blot method	^1^H NMR, DLS, TEM, RT-PCR	[46]
PTX–CYDs complexes	β-CD (1) 2,6-Dimethyl-β-CD (2) 2,3,6-trimethyl-β-CD (3)	DU145 -Prostate cancer	Freeze drying	FTIR, H NMR, DSC, Molecular modeling study using: 1. Monte Carlo using Macro Model package 2. Quasi-flexible docking using MOLINE	[48]
BPEI-SPION NPs	β-cyclodextrin	MCF-7, CT26, HeLa Breast cancer, cervical carcinoma, and colon cancer	Freeze-thaw method	^1^H NMR, TGA, TEM,	[51]

## Data Availability

Not applicable.
